# Identification of a stress-sensitive endogenous opioid-containing neuronal population in the paranigral ventral tegmental area

**DOI:** 10.1038/s41386-025-02292-z

**Published:** 2025-12-20

**Authors:** Carrie Stine, Amanda L. Pasqualini, Ananya S. Achanta, Joseph C. Johnson, Sanjana Jadhav, David J. Marcus, Michael R. Bruchas

**Affiliations:** 1 Center for the Neurobiology of Addiction, Pain and Emotion, Departments of Anesthesiology and Pharmacology, University of Washington, Seattle, WA USA; 2 Molecular and Cellular Biology, University of Washington School of Medicine, Seattle, WA USA

**Keywords:** Stress and resilience, Reward

## Abstract

Nociceptin/orphanin FQ (N/OFQ), an endogenous opioid neuropeptide, and its G-protein coupled receptor NOPR have been implicated in motivation, feeding behaviors, and aversion. Stress-induced dysfunction in these states is central to the development of numerous psychiatric disorders, and the N/OFQ-NOPR system’s role in reward- and stress-related responses has driven broad interest in NOPR as a therapeutic target for anxiety and depression. However, the impact of stress on N/OFQ signaling in the context of its influence on discrete midbrain reward circuitry remains unknown. To this end, we focused on a possible candidate population of N/OFQ neurons in the paranigral ventral tegmental area (pnVTA^PNOC^) that have been shown to act locally on NOPR-containing VTA dopamine neurons to suppress motivation. Here we report and characterize pnVTA^PNOC^ sensitivity during exposure to a diverse range of stressors. Our results indicate that pnVTA^PNOC^ neurons become recruited during exposure to a variety of acute stressor types, suggesting that this N/OFQ population in the pnVTA could act as a critical bridge between stress and motivation.

## Introduction

Stress exposure is a major risk factor in the development of addiction, relapse susceptibility, anxiety, and mood disorders, all of which collectively impose a staggering global health burden [1–4]. While these disorders are vastly diverse, they all commonly involve the emergence of anhedonia and atypical motivation, indicating that the neurobiological mechanisms driving functional reward-related behaviors are highly susceptible to disruption in these disease states [5–9]. Understanding the circuitry and neurobiological substrates central to reward processing that become altered by stress is a critical first step toward identifying viable therapeutic targets with improved function.

The mesolimbic pathway, comprised of dopaminergic projections from the ventral tegmental area (VTA) to the nucleus accumbens (NAc), plays a central role in processing and responding to reward [10, 11]. Converging animal [12–14] and human studies [15–18] have demonstrated that acute stress impacts neural activity within mesolimbic circuitry. In the VTA specifically, stress is generally found to have a net effect of suppressing VTA dopamine (DA) neuron activity [19, 20]. Recent studies have also expanded on the VTA’s molecular complexity by revealing diverse neuropeptide subpopulations, released both by the VTA itself and by upstream inputs that have significant influence over this critical reward circuitry [21–24].

Among these subpopulations, neurons in the paranigral nucleus of the VTA (pnVTA) enriched with the endogenous opioid peptide nociceptin/orphanin FQ (N/OFQ) have recently emerged as key regulators of motivated behavior [25]. Our group previously reported that activation of these N/OFQ-expressing pnVTA neurons (pnVTA^PNOC^ neurons) suppresses motivated reward-seeking behavior and drives aversive responses. Notably, N/OFQ signaling through its cognate G-protein coupled receptor NOPR, which is largely expressed on VTA DA neurons [26], negatively regulates dopamine tone [27], paralleling the effects of stress. Despite widespread implications of N/OFQ in stress responses [28], whether stress impacts this particular pnVTA^PNOC^ population which is critically situated to regulate motivation and reward-related behaviors remains unexplored.

Here, we employed in vivo calcium imaging with the genetically-encoded calcium indicator GCaMP to monitor pnVTA^PNOC^ neuronal dynamics during exposure to physical, environmental, and predatory forms of stress. These findings contribute to a growing understanding of VTA circuitry in stress processing and identify a unique role of pnVTA N/OFQ neurons as a tenable bridge underlying stress regulation of motivated behavior.

## Materials and methods

### Animals

Adult (18–35 g, 3–6 months old) male and female *Pnoc*-IRES-Cre (PNOC-Cre) mice were group housed in the animal facility at 22–24 °C on a 12 h/12 h reverse light/dark cycle (9:00 AM lights off) in ventilated cages with ad libitum access to standard chow and water. All animals were monitored for health status daily and before experimentation for the entirety of the study. Animal procedures were approved by the Animal Care and Use Committee of the University of Washington and conformed to US National Institutes of Health guidelines. All resources are listed in Table S1.

### Stereotaxic surgery

All coordinates, viruses, and volumes for experiments are listed in Table S2. After acclimating to the holding facility for at least seven days, mice were anaesthetized in an induction chamber (1–4% isoflurane) and placed into a stereotaxic frame (Kopf Instruments, model 1900) where they were maintained at 1–2% isoflurane. A blunt needle syringe (86200, Hamilton Company) was used to deliver virus at a rate of 100 nL/min in the pnVTA. An optic fiber (400 µm core, 2.5 mm ferrule, Doric) was slowly lowered to 0.05 mm above the injection site and secured using MetaBond (C & B Metabond). A stainless-steel head-ring was also secured on animals undergoing air puff to allow for head-fixation. Animals were allowed to recover from surgery for a minimum of 3 weeks before any behavioral testing, permitting optimal viral expression.

### Fiber photometry recordings

Fiber photometry studies were completed as described previously [29] (see Supplementary Methods). In brief, GCaMP6s fluorescence was excited using a 470 nm LED (Ca^2+^-dependent signal) and a 405 nm LED (isosbestic control, Ca^2+^-independent signal). LED intensities were set to 30 µW at the optic fiber tip. GCaMP6s emissions were filtered (525 ± 25 nm), detected with a photoreceiver, and recorded by a real-time processor.

### In vivo animal experiments

All animal behaviors were performed within a sound-attenuated room maintained at 23 °C at least one week after habituation to the holding room. Animals were handled for a minimum of three days prior to experimentation and were habituated to fiber photometry patch cord attachment to their fiber implants. For all experiments, mice were brought into the experimental room and allowed to acclimate to the space for at least 30 min prior to any testing. All experiments were conducted in red light to accommodate the reverse light cycle schedule, unless otherwise stated. All sessions were video recorded.

### Behaviors

#### Cued foot shock

Mice were placed in Med Associates Fear Conditioning Chambers (NIR-022MD) which consisted of a 29.5 × 23.5 × 21 cm chamber with a conductive grid floor lit by infrared light and contained within a soundproof box. Mice were exposed to ten 10 s tones co-terminating with a 2 s 0.5 mA shock with a variable inter-trial interval (VITI) of 45–90s.

#### Tail lift

Mice were placed in a 10” x 10” clear acrylic box illuminated by a dim, diffuse white light ( ~ 30 lux). Mice were suspended by the tail four times for 20 s with a VITI of 120–180s. All suspensions were made to the same height.

#### Air puff

Four days prior to testing, mice were habituated to head-fixation on the OHRBETS platform as described previously [29, 30]. A fixed, solenoid-controlled O_2_ valve was positioned above the animal’s left whiskers. Mice were exposed to fifteen 0.1 s 20 PSI air puffs with a VITI of 45–75s. Solenoid opening (Parker, 003-0257-900) was controlled using an Arduino Mega 2560 REV3 (Arduino) and custom Arduino programs.

#### Looming

Mice were placed in a white-walled plexiglass arena (50 × 50 cm) illuminated by a diffuse white light ( ~ 80 lux) and allowed to roam freely. Looming was simulated four times via a posterboard blocking the arena’s overhead lighting (arena illumination reduced to ~25 lux) for 1–2s, with an ITI of 120 s.

#### Odor delivery

Mice were placed in a polyethylene chamber approximately 12 × 12 x 24 cm where air was continuously vacuumed out at a rate of 2 L/min [31]. To minimize odor release into the room, the chamber was placed in a fume hood and vacuumed air was passed through a carbon filter. Odors (2% 2MT or 2% peppermint oil, in separate sessions) were delivered four times per session for 30 s periods with a VITI of 120–180s.

#### Open field test (OFT)

OFT was completed as described previously [31, 32] in a white-walled plexiglass arena (50 × 50 cm) illuminated by a white light ( ~ 200 lux). Center zone was defined as the middle 50% of the arena size. Mice were allowed to roam the arena freely for 30 min.

#### Elevated zero maze (EZM)

EZM was completed as described previously [31, 32] in a circular maze (Harvard Apparatus) with a 200 cm circumference comprised of four 50 cm sections (two open and two closed ‘arms’), elevated 50 cm above the floor illuminated by a white light ( ~ 25 lux). The maze path was 4 cm wide with a 0.5 cm lip on each open arm and 17 cm walls on each closed arm. Mice were positioned head-first into a closed arm and allowed to roam the maze freely for 7 min.

### Tissue preparation and immunohistochemistry (IHC)

Unless otherwise stated, animals were transcardially perfused with 0.1 M phosphate-buffered saline (PBS) followed by 40 mL 4% paraformaldehyde. Brains were dissected and post-fixed in 4% paraformaldehyde overnight and then transferred to 30% sucrose solution for cryoprotection. Brains were sectioned at 30 µm on a microtome and stored in 0.1 M phosphate buffer at 4 °C prior to immunohistochemistry and tracing experiments. Immunohistochemistry was completed as described previously [29, 33, 34] (see Supplementary Methods). For behavioral cohorts, viral expression and optic fiber placements were evaluated before inclusion in the presented datasets.

### Fiber photometry analysis

Fiber photometry data were analyzed as described previously [29]. In brief, custom MATLAB scripts were used to normalize signal by detrending bleaching decay and correcting for motion artifact using the isosbestic trace. Normalized traces were extracted in windows surrounding the onset of relevant behavioral events (tail lift, odor, shock, air puff, looming, open arm entry, center entry), z-scored relative to the mean and standard deviation of a 10-s baseline period preceding each event window, and then averaged.

### Behavioral scoring

For foot shock and looming video recordings, behavior annotations were conducted manually by a blinded investigator to avoid bias in scoring sex differences. A trial was categorized as “freeze” if mice were immobile for at least 2 s following stimulus onset, whereas a trial was categorized as “flight” if mice fled from their original position with enhanced velocity within 5 s of stimulus onset. Trials not classified as either freeze or flight were labeled as “neither.”

### Statistical analyses

Statistical analyses were performed as indicated (see Supplementary Methods) in GraphPad Prism 9 and MATLAB 9.9 (MathWorks). For spontaneous behaviors where experimenters did not control the timing of each trial (open field test, elevated zero maze), any trials that occurred less than 10 s after a directly preceding trial were removed to prevent signal contamination within the baseline period. No other data were excluded from analyses. All data are expressed as mean ± SEM unless otherwise specified.

## Results

### pnVTA^PNOC^ neurons exhibit sustained activity throughout acute stress exposure across multiple stress conditions

**Fig. 1:**
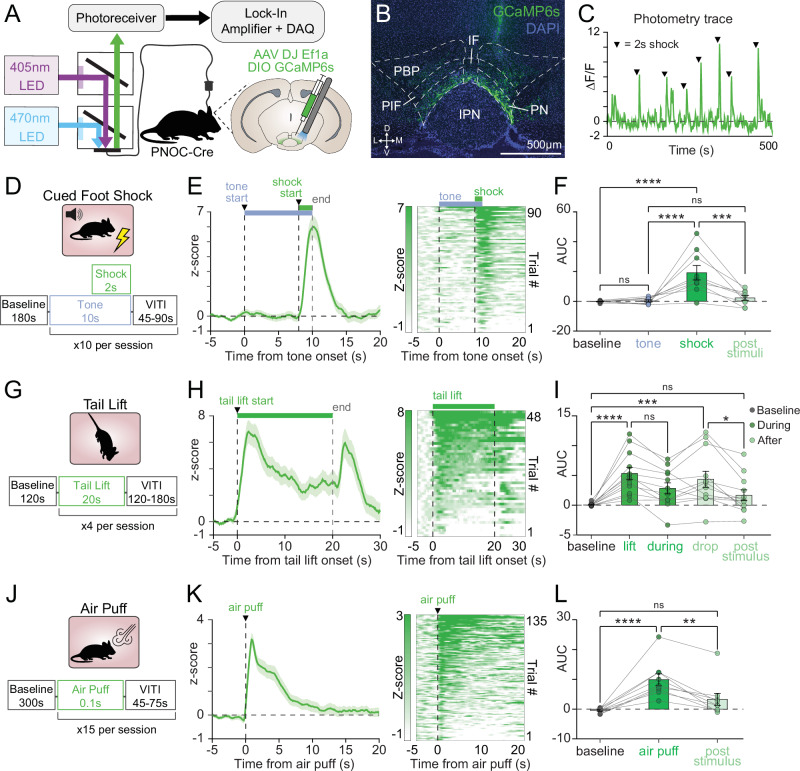
pnVTA^PNOC^ neurons are activated during exposure to acute stressors. **A** Fiber photometry schematic and cartoon of DIO-GCaMP6s (GCaMP6s) viral injection and fiber implant in the pnVTA of PNOC-Cre mice. **B** Representative coronal image showing DAPI (blue) and GCaMP6s (green) expression in pnVTA. **C** Representative trace of GCaMP6s ∆F/F fluorescence throughout a cued foot shock session. Black arrows are aligned with foot shock onset. **D** Trial structure for a cued foot shock session (10 s tone co-terminating with 2 s 0.5 mA shock). **E** Left: Averaged trace of pnVTA^PNOC^ GCaMP6s activity during epoch surrounding tone-cued foot shock, aligned to tone onset. Right: Intensity-sorted heat map of GCaMP6s fluorescence during same epoch, each row correspond to a trial in the averaged trace (left). (*N* = 9 mice). **F** Area under the curve (AUC) for averaged traces from (**E**), calculated over 8-s intervals surrounding cued-foot shock events. GCaMP6s signal increases in response to shock but not tone (one-way repeated-measures ANOVA main effect of time [F3,24 = 14.26, *p* < 0.0001]. Tukey’s multiple comparisons test [****p < 0.0001, ***p = 0.0003], *N* = 9 mice). **G**–**I** Same as (**D**–**F**) but for pnVTA^PNOC^ GCaMP6s activity during 20 s tail lift. Activity averaged in 5-s intervals surrounding each tail lift shows increases first during tail lift and again when animal is lowered to the ground (one-way repeated-measures ANOVA main effect of time [F4,44 = 10.94, *p* < 0.0001]. Tukey’s multiple comparisons test [****p < 0.0001, ***p = 0.0002, *p = 0.0342], *N* = 12 mice). **J**–**L** Same as (**D**–**F**) but for pnVTA^PNOC^ GCaMP6s activity during acute air puff (0.1 s, 20 PSI). Activity averaged in 5-s intervals surrounding each air puff (one-way repeated-measures ANOVA main effect of time [F2,14 = 13.99, *p* = 0.0005]. Tukey’s multiple comparisons test [****p < 0.0001, **p = 0.0044], *N* = 9 mice). All data represented as mean ± SEM.

N/OFQ-containing neurons in the pnVTA (pnVTA^PNOC^ neurons) act to suppress motivated behaviors. Stress is also known to disrupt motivation, but despite evidence linking N/OFQ with stress, the impact of stress on the activity of this population remains unknown. Importantly, the effects of stress exposure on motivation can vary depending on the form and duration of the stressor. To evaluate the effects of diverse stress conditions on pnVTA^PNOC^ activity, we injected PNOC-Cre mice with a Cre-dependent GCaMP6s (AAV-DJ-Ef1a-DIO-GCaMP6s) and implanted optic fibers in the paranigral ventral tegmental area (pnVTA) to record the calcium activity of pnVTA N/OFQ neurons (pnVTA^PNOC^) during exposure to a variety of stressful stimuli (Fig. 1A, B). At 3–4 weeks post injection we detected robust, transient activation of pnVTA^PNOC^ neurons in response to a mild foot shock (Fig. 1C–F, one-way repeated-measures ANOVA main effect of time F3,24 = 14.26, *p* < 0.0001; Tukey’s baseline vs shock *p* < 0.0001), a 20-s tail lift (Fig. 1G–I, one-way repeated-measures ANOVA main effect of time F4,44 = 10.94, *p* < 0.0001; Tukey’s baseline vs lift *p* < 0.0001), and a brief 0.1 s air puff delivered to the whiskers (Fig. 1J–L, one-way repeated-measures ANOVA main effect of time F2,16 = 18.05, *p* < 0.0001; Tukey’s baseline vs puff *p* < 0.0001).

Interestingly, pnVTA^PNOC^ activity was time-locked with the duration of the stressor. Activity returned to baseline levels following the offset of the 2 s foot shock (Fig. 1F, Tukey’s baseline vs post-stimuli *p* = 0.8908) or 0.1 s air puff (Fig. 1L, Tukey’s baseline vs post-stimulus *p* = 0.1113) but remained elevated throughout the 20 s tail suspension (Fig. 1I, Tukey’s baseline vs during *p* = 0.0275). Across these stressors we did not detect sex-dependent effects on pnVTA^PNOC^ activity during stress (Supplementary Fig. 1A–C, two-way repeated-measures ANOVA main effect of sex, foot shock: F1,7 = 2.699, *p *= 0.1444; tail lift: F1,10 = 0.8557, *p* = 0.3767; air puff: F1,7 = 0.2339, *p* = 0.6434), although foot shock did elicit a larger response in females (Supplementary Fig. 1A, two-way repeated-measures ANOVA interaction between time and sex F3,21 = 5.505, *p* = 0.006; Tukey’s shock male vs female *p* = 0.0003). Notably, pnVTA^PNOC^ neurons were not activated in response to the 10-s tone that preceded each foot shock (Fig. 1E, F, Tukey’s baseline vs. tone *p* = 0.9993), suggesting that pnVTA^PNOC^ neurons have selective sensitivity to stress rather than simply responding indiscriminately to any salient stimuli.

### Stressful environmental cues elicit pnVTA^PNOC^ neuron activation during exploration

**Fig. 2:**
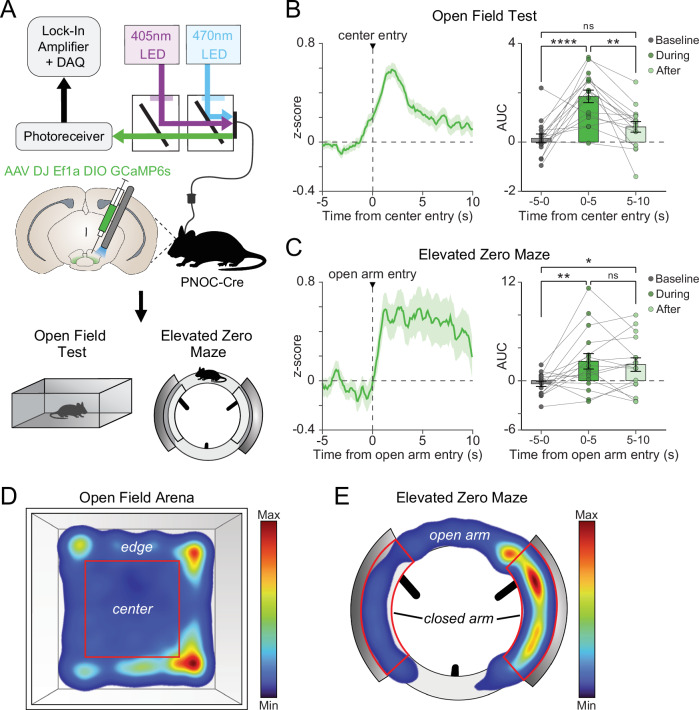
Anxiogenic exploratory behaviors drive pnVTA^PNOC^ activity. **A** Cartoon of DIO-GCaMP6s (GCaMP6s) injection and fiber implant into pnVTA of PNOC-Cre mice. GCaMP6s activity was recorded during open field test (OFT) and elevated zero maze (EZM). **B** Left: Averaged traces of pnVTA^PNOC^ GCaMP6s activity during high-anxiety epochs of the OFT, aligned to entries into the center zone of the open field arena. Right: Area under the curve (AUC) for averaged traces (left) calculated over 5-s intervals surrounding center zone entry. GCaMP6s activity increases during and immediately after center entry (one-way repeated-measures ANOVA main effect of time [F2,30 = 16.18, *p* < 0.0001]. Tukey’s multiple comparisons test [****p < 0.0001, **p = 0.0011], *N* = 16 mice). **C** Same as (**B**) but for pnVTA^PNOC^ GCaMP6s activity during high-anxiety epochs of the EZM, aligned to entries into either open arm of the maze (one-way repeated-measures ANOVA main effect of time [F2,30 = 6.305, *p* = 0.0052]. Tukey’s multiple comparisons test [**p = 0.007, *p = 0.0233], *N* = 16 mice). **D** Heat map from a representative animal showing proportion of time spent in each area of the open field arena. Heat map shows more time spent around the edge than in the center. **E** Same as (**D**) but showing proportion of time spent in each area of the elevated zero maze. Heat map shows more time spent in the closed arms than in the open arms. All data represented as mean ± SEM.

We next evaluated pnVTA^PNOC^ dynamics during innately anxiogenic exploratory behaviors (Fig. 2A). In the open field test (OFT), we detected a significant increase in calcium activity as animals transitioned from the ‘safe’ edge of the arena to the open, ‘risky’ center (Fig. 2B, D, one-way repeated-measures ANOVA main effect of time F2,30 = 16.18, *p* < 0.0001; Tukey’s baseline vs center entry *p* < 0.0001). We observed a similar increase in the elevated zero maze (EZM) as animals entered the unprotected open arms of the maze (Fig. 2C, E, one-way repeated-measures ANOVA main effect of time F2,30 = 6.305, *p* = 0.0052; Tukey’s baseline vs open arm entry *p* = 0.007). No sex-dependent effects were identified in either OFT or EZM (Supplementary Fig. 1D, E, two-way repeated-measures ANOVA main effect of sex, OFT: F1,14 = 0.0007, *p* = 0.9794; EZM: F1,14 = 1.058, *p* = 0.3212). These findings demonstrate that pnVTA^PNOC^ neurons are also engaged by innately stressful environmental stimuli. Taken together, our results indicate that multiple forms of acute stress elicit robust activation of N/OFQ-containing pnVTA neurons.

### Predator odor stress engages pnVTA^PNOC^ neurons in both sexes, while predator looming stress elicits responses only in males

**Fig. 3:**
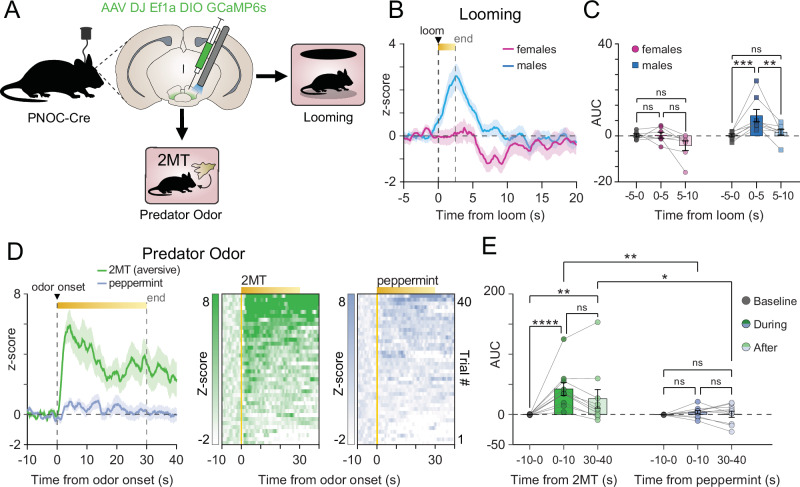
pnVTA^PNOC^ neuron activity is recruited in response to predatory threat. **A** Cartoon of DIO-GCaMP6s (GCaMP6s) injection and fiber implant into pnVTA of PNOC-Cre mice. GCaMP6s activity was recorded during looming or exposure to predator odor. **B** Averaged traces of pnVTA^PNOC^ GCaMP6s activity for males (blue, *N* = 5 mice) and females (magenta, *N* = 7 mice) aligned to looming onset. **C** Area under the curve (AUC) for averaged traces from B for females (left, magenta) and males (right, blue), calculated over 5-s intervals. GCaMP6s activity increases during and immediately after looming in males, but not females (two-way repeated-measures ANOVA main effect of time [F2,26 = 9.383, *p* = 0.0009], main effect of sex [F1,13 = 8.796, *p* = 0.0109], interaction of time x sex [F2,26 = 4.728, *p* = 0.0177]. Tukey’s multiple comparisons test [***p = 0.0004, **p = 0.0027], *N* = 8 males, 7 females). **D** Left: Averaged traces of pnVTA^PNOC^ GCaMP6s activity surrounding 30-s exposure to either predator odor (2% 2MT, green) or a control non-predator odor (2% peppermint oil, blue). Right: Intensity-sorted heat map of GCaMP6s fluorescence during same epoch, each row correspond to a trial in the averaged trace (left). (*N* = 10 mice). **E** AUC for averaged traces from D calculated over 10-s intervals surrounding exposure to either the 2MT predator odor (left, green) or the peppermint oil (right, blue). pnVTA^PNOC^ GCaMP6s activity increases during 2MT but not peppermint oil exposure (two-way repeated-measures ANOVA main effect of time [F4,72 = 6.173, *p* = 0.0003], main effect of odor [F1,18 = 4.896, *p* = 0.0401], interaction of time x odor [F4,72 = 4.041, *p* = 0.0052]. Tukey’s multiple comparisons test, 2MT over time [****p < 0.0001, **p = 0.0032], 2MT vs peppermint [**p = 0.0025, *p = 0.0419], *N* = 10 mice per group). All data represented as mean ± SEM.

We further characterized the stress sensitivity of pnVTA^PNOC^ neurons by recording calcium activity following different predatory stressors (Fig. 3A). When exposed to a looming stimulus that mimics the threat of an overhead predator, male mice displayed a significant increase in pnVTA^PNOC^ calcium activity while females did not (Fig. 3B,C, two-way repeated-measures ANOVA main effect of sex F1,13 = 8.796, *p* = 0.0109; Tukey’s male baseline vs loom *p* = 0.0004, female baseline vs loom *p* = 0.9979).

We also assessed pnVTA^PNOC^ calcium activity dynamics in response to an aversive predator odor (2% 2MT, a predator urine derivative [35]) and a non-aversive novel odor (2% peppermint oil), which served as a control for salience (Fig. 3D). The aversive 2MT predator odor evoked a robust, sustained increase in calcium activity, whereas the non-aversive peppermint odor elicited no response (Fig. 3E, 2MT: one-way repeated-measures ANOVA F4,36 = 6.007, *p* = 0.0008; peppermint oil: one-way repeated-measures ANOVA F4,36 = 0.5098, *p* = 0.7288). These data are consistent with our initial findings that pnVTA^PNOC^ neurons are selectively activated by stressful stimuli and reveal a potential sex-dependent circuit level effect in response to certain predatory stressors, in male mice.

### Threat-response strategies were similar between male and female mice, but female mice spent more time freezing after looming

**Fig. 4:**
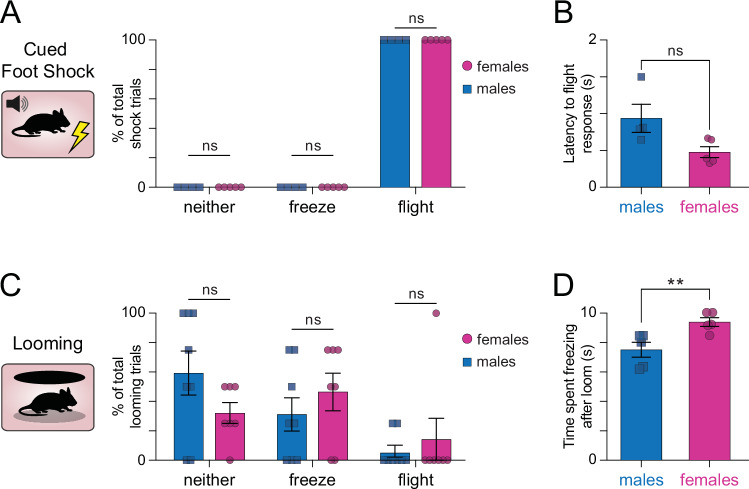
Male and female mice employ similar behavioral strategies in response to shock and looming, but females spend more time freezing during looming. **A** Percentage of total shock trials (10 per session) where each male (blue, square) or female (magenta, circle) mouse showed behavioral responses categorized as either freeze, flight, or neither during foot shock (100% flight response, SD = 0 for all behavior types. *N* = 5 females, 4 males). **B** Trial averaged latency to flight response following foot shock (two-tailed Mann–Whitney test, males vs females *p* = 0.0635, *N* = 5 females, 4 males). **C** Percentage of total looming trials (4 per session) where each male (blue, square) or female (magenta, circle) mouse showed behavioral responses categorized as either freeze, flight, or neither during the loom (two-way repeated-measures ANOVA main effect of behavior [*F*_*2,39*_ = 5.457, *p* = 0.0081], main effect of sex [*F*_*1,39*_ = 0.000, *p* > 0.9999], interaction of behavior x sex [*F*_*2,39*_ = 0.9989, *p* = 0.3775]. Tukey’s multiple comparisons test male vs female in behavior type neither: [*p* = 0.2586], freeze: [*p* = 0.4956], and flight: [*p* = 0.6491]. *N* = 7 females, 8 males). **D** Trial averaged time spent freezing in the 10-s window beginning at loom onset for animals that had a freeze response to looming (two-tailed Mann–Whitney test, males vs females *p* = 0.0079, *N* = 5 females, 5 males). All data represented as mean ± SEM.

During foot shock, all mice of both sexes employed a ‘flight’ escape response following the onset of the shock (Fig. 4A, 100% flight response, SD = 0, *N* = 4 males, 5 females). Males and females also had similar latencies to initiation of the flight behavior, although there was a slight but non-significant trend toward faster response time in females (Fig. 4B, two-tailed Mann–Whitney test, *p* = 0.0635). There were no detectable sex-dependent differences in behavioral response during the cue period that preceded each foot shock trial (Supplementary Fig. 2, two-way repeated-measures ANOVA main effect of sex on behavioral response *F*_1,21_ = 0.000, *p* > 0.999; two-tailed Mann Whitney test on time spent freezing to tone, males vs females *p* = 0.1905). These results suggest that the sex-dependent difference in pnVTA^PNOC^ potentiation during shock is not accompanied by notable differences in behavior between males and females.

During looming stress, although mice had more overall variation in which strategy they employed in response to the stressor, there were similar proportions of male and female mice that opted for flight, freeze, or neither strategy (Fig. 4C, two-way repeated-measures ANOVA main effect of sex on behavioral response *F*_1,39_ = 0.000, *p* > 0.999;). During looming, the primary difference in pnVTA^PNOC^ signal between sexes was detected during the first 10 s after the loom. Since freezing was the more prevalent threat-response for this behavior compared to flight, we quantified the total time each sex spent freezing during this 10-s time period (Fig. 4D). Interestingly, female mice spent more time freezing during this time window compared to male mice (Fig. 4D, two-tailed Mann Whitney test on time spent freezing to loom, males vs females *p* = 0.0079). These findings indicate that the lack of pnVTA^PNOC^ activity we observed during looming in females but not males is accompanied by a sex-dependent difference in freezing behavior.

### pnVTA^PNOC^ activation does not sensitize to repeated stress exposure across an acute exposure session

**Fig. 5:**
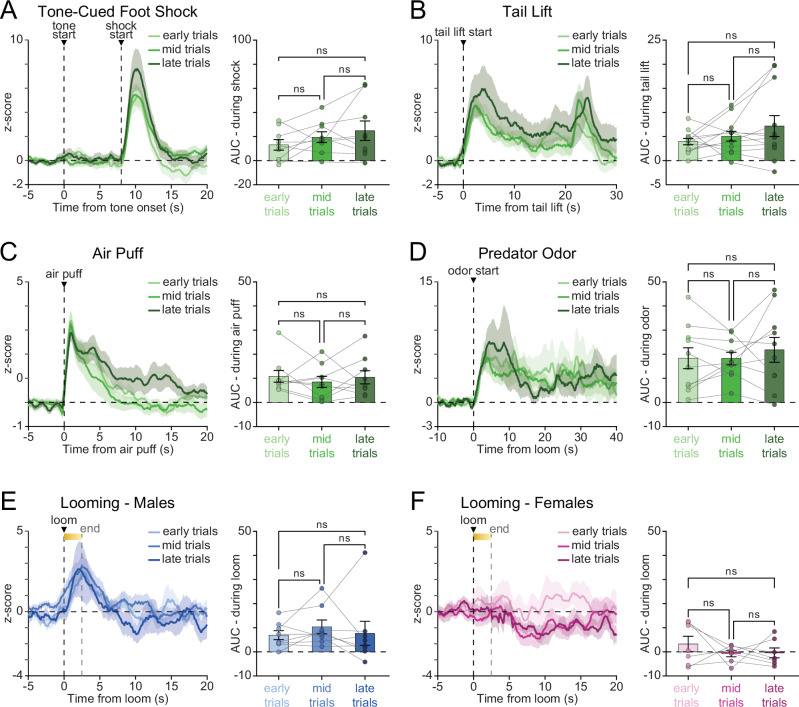
pnVTA^PNOC^ activation does not sensitize to repeated exposure of a stressful stimulus. **A** Left: Averaged traces of pnVTA^PNOC^ GCaMP6s activity during epoch surrounding tone-cued foot shock, aligned to tone onset. Trials were grouped by when they occurred during the session, with early trials (light green) in the first third, mid trials (medium green) in the second third, and late trials (dark green) occurring in the final third of the session. Right: Area under the curve (AUC) for averaged traces from left panel, calculated over the 8-s window following foot shock. AUC was similar across early, mid, and late trials (one-way repeated-measures ANOVA main effect of time *F*_2,16_ = 2.403, *p* = 0.1223. Tukey’s multiple comparisons test, ns = non-significant. *N* = 9 mice). **B**–**F** Same as (**A**) but for pnVTA^PNOC^ GCaMP6s activity during B 20 s tail lift (one-way repeated-measures ANOVA main effect of time *F*_2,22_ = 1.803, *p* = 0.1884. Tukey’s multiple comparisons test, ns = non-significant. *N* = 12 mice), C 0.1 s air puff (one-way repeated-measures ANOVA main effect of time *F*_2,16_ = 0.5601, *p* = 0.5820. Tukey’s multiple comparisons test, ns = non-significant. *N* = 9 mice), **D** predator odor (one-way repeated-measures ANOVA main effect of time *F*_2,18_ = 0.4393, *p* = 0.6512. Tukey’s multiple comparisons test, ns = non-significant. *N* = 10 mice), E looming in male mice (one-way repeated-measures ANOVA main effect of time *F*_2,14_ = 0.4527, *p* = 0.6449. Tukey’s multiple comparisons test, ns = non-significant. *N* = 8 mice), and **F** looming in female mice (one-way repeated-measures ANOVA main effect of time *F*_2,12_ = 1.180, *p* = 0.3405. Tukey’s multiple comparisons test, ns = non-significant. *N* = 7 mice). For data in (**B**–**F**), AUC was calculated over the 5-s window following stress exposure onset. All data represented as mean ± SEM.

Since we observed differences in the magnitude of pnVTA^PNOC^ activity across different types of stressors, we next examined whether activation of these neurons became sensitized by repeated exposure to the same stressor. For each of the experimentally-evoked stressful stimuli that we tested (foot shock, tail lift, air puff, predator odor, and looming), mice experienced multiple presentations of the stressor within a single experimental session. For each of these stress types, we divided each experimental session into three time periods, early, mid, and late, and then evaluated pnVTA^PNOC^ activity during trials that occurred within each time period (Fig. 5).

We found that the magnitude of pnVTA^PNOC^ neuron responses remained consistent across early, mid, and late trials for all tested stressors, including tone-cued foot shock (Fig. 5A, one-way repeated-measures ANOVA main effect of time *F*_2,16_ = 2.403, *p* = 0.1223), 20 s tail lift (Fig. 5B, one-way repeated-measures ANOVA main effect of time *F*_2,22_ = 1.803, *p* = 0.1884), 0.1 s air puff (Fig. 5C, one-way repeated-measures ANOVA main effect of time *F*_2,16_ = 0.5601, *p* = 0.5820), predator odor (Fig. 5D, one-way repeated-measures ANOVA main effect of time *F*_2,18_ = 0.4393, *p* = 0.6512), and looming (Fig. 5E, F, one-way repeated-measures ANOVA main effect of time males: [*F*_2,14_ = 0.4527, *p* = 0.6449], females: [*F*_2,12_ = 1.180, *p* = 0.3405]). In conclusion, these findings indicate that pnVTA^PNOC^ activation is stable across repeated exposure to a given stressor occurring within an acute ( < 30 min) time frame.

## Discussion

The aim of this study was to characterize how stress exposure impacts pnVTA^PNOC^ neuron activity. We demonstrate that pnVTA^PNOC^ neurons are selectively activated in response to stress rather than salience in a primarily non-sex-dependent manner, except during looming predator stress where activity increased in males only. Our findings reveal new insights into a pathway by which stress interfaces with a neuropeptide subpopulation known to direct motivated reward-seeking behaviors through its influence on mesolimbic circuitry. We also show that pnVTA^PNOC^ neurons do not sensitize with acute, repeated exposure to the same stressor, but do have varying sensitivity to different stressors, with physical and predator-based stimuli producing a larger dynamic response than the innately stressful environmental cues experienced during exploratory behaviors. Whether these differences in dynamics are related to the perceived valence of the stressful stimulus will be an important follow-up for further study.

In this study we recorded changes in calcium activity via GCaMP6s fluorescence as a proxy for pnVTA^PNOC^ neuron activity. It is important to note that although calcium activity can serve as an indirect measure of neuron activity, our recordings do not directly measure N/OFQ release within the VTA and therefore do not necessarily reflect recruitment of endogenous N/OFQ signaling. The recent development of biosensors specific to a wide array of neuromodulators and neuropeptides, including a sensor for N/OFQ [29], have opened up new avenues for direct detection of neuromodulator release and should be used in future studies to examine N/OFQ dynamics during the stress response within the pnVTA neuronal population.

Although the looming behavior was not formally cued, the timing of signal onset in pnVTA^PNOC^ neurons slightly precedes the initiation of the looming trial (Fig. 2B). This suggests that the mice may have detected inadvertent environmental cues (e.g. movement of the researcher to initiate a trial) in anticipation of the upcoming loom trial, which is a limitation to consider when interpreting the temporal precision of this specific behavior.

N/OFQ has been implicated in the stress response, but the observed effects of stress on the N/OFQ-NOPR system vary widely both across the brain and depending on the form and duration of stress exposure [28, 36]. Prior studies have also reported notable sex differences in rats, where stress-induced changes to N/OFQ expression were more prominent in males [37, 38]. While most of the stressors we evaluated in this study evoked similar pnVTA^PNOC^ responses in males and females, the activity during looming that occurred exclusively in male mice mirrors these prior findings where a stress-evoked change in N/OFQ was only seen in males. On the contrary, we observed a potentiation of pnVTA^PNOC^ activation during foot shock in females relative to males (Supplementary Fig. 1), suggesting that sex-dependent effects on how N/OFQ neurons respond to stress can also extend to females. This potentiation during foot shock was not accompanied by any detectable behavioral differences between sexes. In contrast, the lack of pnVTA^PNOC^ response in females during looming was accompanied by an increase in freezing time compared to males, suggesting a sex-dependent effect on the presence but not the magnitude of pnVTA^PNOC^ activation in response to stress (Figs. 3 and 4). Given the lack of a sex-dependent difference in the behavioral response to foot shock, it would be interesting to determine whether individual heterogeneity in terms of an animal’s resilience or vulnerability to stress shapes the magnitude of pnVTA^PNOC^ neuron response during stress exposure.

The N/OFQ-NOPR system has been closely linked with anhedonia and changes to motivated behavior (for a summary see Gavioli and Calo, 2013). It is well established that stress is a prominent factor in the pathophysiology and development of anhedonia and motivational deficits, but the specific circuit-level mechanisms by which stress alters motivated behaviors are less understood. In previous work, we reported that activation of pnVTA^PNOC^ activity suppresses effort-based reward seeking in mice [25], suggesting a mechanism by which N/OFQ activity in this circuit may regulate motivation. Our findings in this study demonstrate that these pnVTA^PNOC^ neurons are also sensitive to acute stress, identifying a potential pathway through which stress could suppress motivation by driving excessive activation of N/OFQ signaling in the pnVTA, and subsequent modulation of dopaminergic tone as previously demonstrated by our group [25]. Further work with specialized genetic approaches is needed to more directly test this hypothesis however, particularly in evaluating whether inhibition of pnVTA^PNOC^ activity can improve chronic stress-induced motivation deficits, and furthermore how NOPR expressing dopamine neuron activity is impacted under these situations.

In our previous work, we found that over-activation of pnVTA^PNOC^ neurons suppressed reward-seeking behavior in mice while pnVTA^PNOC^ inhibition enhanced motivation [25]. This suggests that N/OFQ neuron activity may be critical for fine-tuning reward-related behavior, and imbalances in either direction of standard expression levels could drive opposing effects on motivation. Our findings here showed enhanced pnVTA^PNOC^ activation in response to stress, thus we would predict that stress-induced activation of this population would contribute to an overall anhedonic phenotype. However, it is important to note that our findings are still limited by the selection of stressors used in this study. Given that some forms of stress exposure have also been shown to exacerbate impulsive behaviors such as drug self-administration [39, 40], further work should investigate if other forms and extended durations of stress have a suppressive effect on pnVTA^PNOC^ activity, in addition to evaluating their effects on motivated behavior.

In conclusion, the findings we report here provide insight into how stress modulates N/OFQ neuron activity within the VTA microcircuitry. These results advance our understanding of how the N/OFQ system interfaces with both stress and motivational deficits within a single circuit, laying the groundwork for further studies to explore stress-related mechanisms for anhedonic behavior in the context of this neuropeptidergic pathway. Future research should examine the long-term effects of chronic stress on pnVTA^PNOC^ activity and evaluate the therapeutic potential of NOPR antagonism within this circuit during motivation in models of stress-induced anhedonia.

## Supplementary information

Supplemental Material

## Data Availability

The datasets generated and analyzed in this study are available from the corresponding author upon reasonable request.
