# Supplementary material for: Identification of a stress-sensitive endogenous opioid-containing neuronal population in the paranigral ventral tegmental area
Source: Neuropsychopharmacology. 2025 Dec 20;51(5):831–9. doi: 10.1038/s41386-025-02292-z (PMC13013705; doi:10.1038/s41386-025-02292-z)
Supplement: Supplementary file 1 — Supplemental Material [file 41386_2025_2292_MOESM1_ESM.pdf]

SUPPLEMENTARY INFORMATION

Supplementary Figures

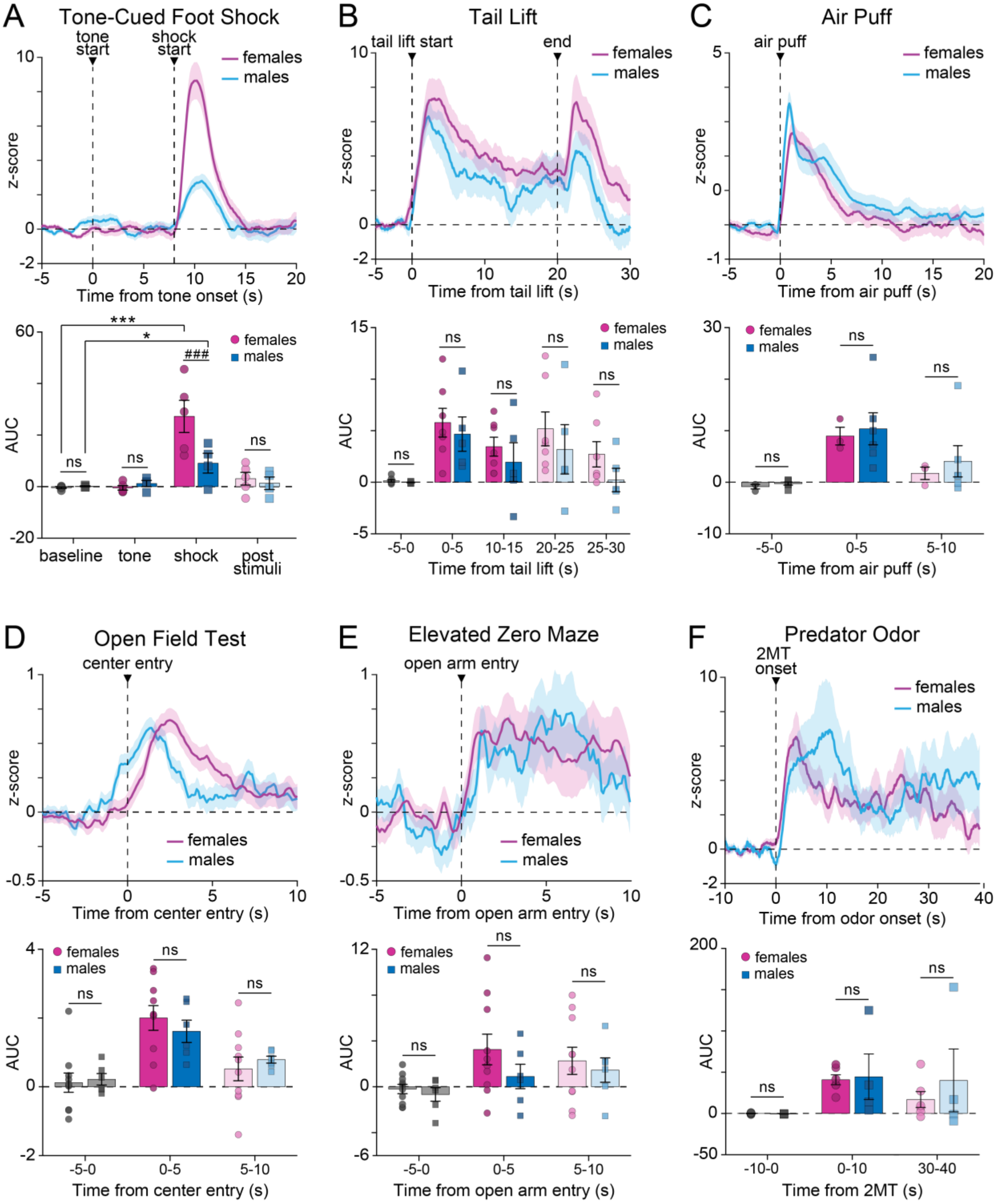

Supplementary Figure 1: Stress engages pnVTA^{PNOC} neurons in both male and female mice.

A Top: Averaged trace of pnVTA^{PNOC} GCaMP6s activity during epoch surrounding tone-cued foot shock, aligned to tone onset in male (blue) and female (magenta) mice. Bottom: Area under the curve (AUC) for averaged traces, calculated over 8-second intervals surrounding cued-foot shock events for males (blue) and females (magenta). GCaMP6s signal increases in response to shock but not tone for both females (one-way repeated-measures ANOVA main effect of time [$F_{3,12}$ = 17.50, p = 0.0001]. Tukey's multiple comparisons test [***p = 0.0003], N = 5 female mice) and males (one-way repeated-measures ANOVA main effect of time [$F_{3,9}$ = 5.424, p = 0.0209]. Tukey's multiple comparisons test [*p = 0.0258], N = 4 male mice). The magnitude of this increase is larger in females than males (two-way repeated-measures ANOVA main effect of time [$F_{3,21}$ = 19.65, p < 0.0001], main effect of sex [$F_{1,7}$ = 2.699, p = 0.1444], interaction of time x sex [$F_{3,21}$ = 5.505, p = 0.006]. Tukey's multiple comparisons test male vs female [$^{###}p$ = 0.0003], N = 5 females, 4 males). **B–F** Same as A for **B** 20s tail lift (two-way repeated-measures ANOVA main effect of sex [$F_{1,10}$ = 0.8557, p = 0.3767], N = 5 males, 7 females), **C** 0.1s air puff (two-way repeated-measures ANOVA main effect of sex [$F_{1,7}$ = 0.2339, p = 0.6434], N = 6 males, 3 females), **D** open field test center entry (two-way repeated-measures ANOVA main effect of sex [$F_{1,14}$ = 0.0007, p = 0.9794], N = 6 males, 10 females), **E** elevated zero maze open arm entry (two-way repeated-measures ANOVA main effect of sex [$F_{1,14}$ = 1.058, p = 0.3212], N = 6 males, 10 females), and **F** predator (2% 2MT) odor (two-way repeated-measures ANOVA main effect of sex [$F_{1,8}$ = 0.1234, p = 0.7345], N = 4 males, 6 females). All data represented as mean ± SEM.

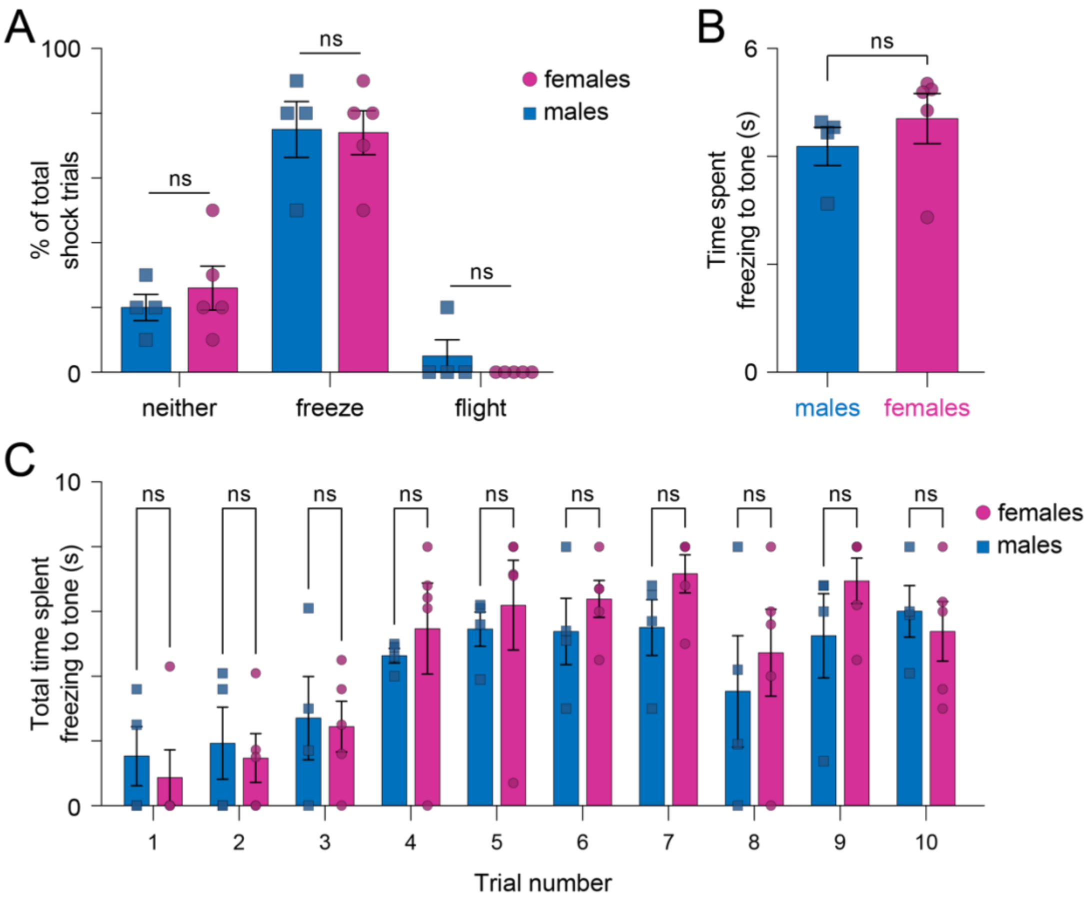

Supplementary Figure 2: Male and female mice exhibit similar behavior during cued tone that precedes shock.

A. Percentage of total trials (10 per session) where each male (blue, square) or female (magenta, circle) mouse showed behavioral responses categorized as either freeze, flight, or neither during the tone cue period preceding each foot shock (two-way repeated-measures ANOVA main effect of behavior [$F_{2,21}$ = 79.28, p < 0.0001], main effect of sex [$F_{1,21}$ = 0.000, p > 0.9999], interaction of behavior x sex [$F_{2,21}$ = 0.4465, p = 0.6458]. Tukey's multiple comparisons test male vs female in behavior type neither: [p = 0.4794], freeze: [p = 0.9056], and flight: [p = 0.5549]. N = 5 females, 4 males). **B.** Quantification of average time each animal spent freezing during the entire 8-second cue period (two-tailed Mann-Whitney test, p = 0.1905, N = 5 females, 4 males). **C.** Quantification of total time spent freezing during the 8-second cue period on a trial-by-trial basis in males vs females (two-way repeated-measures ANOVA main effect of trial [$F_{9,63}$ = 7.869, p < 0.0001], main effect of sex [$F_{1,7}$ = 0.7014, p = 0.4300], interaction of trial x sex [$F_{9,63}$ = 0.4525, p = 0.9005]. Tukey's multiple comparisons test male vs female, ns = non-significant. N = 5 females, 4 males). All data represented as mean ± SEM.

Table S1: Key Resources

REAGENT OR RESOURCE	SOURCE	IDENTIFIER
**Antibodies**		
Chicken-anti-GFP 1:2000	Abcam	Ab13970
Goat anti-chicken IgY, Alexa Fluor 488	Abcam	Ab150169
**Bacterial and Virus Strains**		
AAV-DJ-Ef1a-DIO-GCaMP6s	Stanford University<br>Gene Vector and Viral Core	GVVC-AAV-91
**Experimental Models: Organisms/Strains**		
Pnoc-IRES-Cre (PNOC-Cre)	The Jackson Laboratory	#034278
**Reagents**		
2-Methylthiazoline (2MT)	Tokyo Chemical Industry Co	M0285
Peppermint oil	Sigma Aldrich	77411

Table S2: Viruses, injection coordinates, and implant types

Experiment	Mouse Line	Virus	Titer	Amount	Coordinates
pnVTA-PNOC stress response (Figures 1–3)	PNOC-Cre	AAV-DJ-Ef1a-DIO-GCaMP6s	3.0e12	300nL	AP -3.25, ML 1.6, DV -4.65 (15° angle)

Supplementary Methods

Fiber photometry

531-Hz sinusoidal LED light (Thorlabs, LED light: M470F3; LED driver: DC4104) was bandpass filtered (470 ± 20nm, Doric, FMC4) to excite GCaMP6s, a 211-Hz sinusoidal LED light (Thorlabs, M405FP1; LED driver: DC4104) was bandpass filtered (405 ± 10nm, Doric, FMC4) to evoke Ca^{2+}-independent isosbestic control emission. LED intensities were measured at the tip of the optic fiber and adjusted to 30μW before each recording. GCaMP6s emission traveled back through the same optic fiber then was bandpass filtered, (525 ± 25nm, Doric, FMC4), detected by a photoreceiver (Doric, DFD_FOA_FC), and recorded by a real-time processor (TDT, RZ5P). For the ChrimsonR stimulation experiments, a 635nm laser was passed through the filter cube at 1mW intensity to deliver red light at the tip of the optic fiber [32].

Fiber photometry analysis

Custom MATLAB scripts were used to detrend decay from bleaching by fitting a 4th degree polynomial function to the raw signal (470nm) and isosbestic (405nm) traces, then dividing by the resulting curve. Then signal was normalized by dividing an LLS fit of the isosbestic trace scaled to the signal. The processed traces were then down-sampled by a factor of 100, smoothed across a rolling 1s window, extracted in windows surrounding the onset of relevant behavioral events (tail lift, odor, shock, air puff, looming, open arm entry, center entry), z-scored relative to the mean and standard deviation of a 10-second baseline period preceding each event window, and then averaged.

Immunohistochemistry

Free-floating brain sections were washed in 0.1 M PBS for 3 x 10 min intervals. Sections were then placed in blocking buffer (0.5% Triton X-100 and 5% natural goat serum in 0.1 M PBS) for 1 hour at room temperature. After blocking buffer, sections were placed in primary antibody (chicken

anti-GFP, 1:2000, Abcam) overnight at 4°C. After 3 x 10 min 0.1 M PBS washes, sections were incubated in secondary antibody (AlexaFluor 488 goat anti-chicken, Abcam) for 2 hours at room temperature, followed by another round of washes (3 x 10 min in 0.1 M PBS, 3 x 10 min in 0.1 M PBS). After immunostaining, sections were mounted and coverslipped with Vectashield HardSet mounting medium containing DAPI (Vector Laboratories) and imaged on a Leica DM6 B microscope.
